# *Epimedium brevicornum* Maxim alleviates diabetes osteoporosis by regulating AGE-RAGE signaling pathway

**DOI:** 10.1186/s10020-025-01152-2

**Published:** 2025-03-15

**Authors:** Shan Shan Lei, Yu Yan Wang, Xiao Wen Huang, Xu Ping Wang, Ming Gao, Bo Li

**Affiliations:** 1 Department of Medicine, Zhejiang Academy of Traditional Chinese Medicine, Hangzhou, Zhejiang 310007 P.R. China; 2 Collaborative Innovation Center of Yangtze River Delta Region Green Pharmaceuticals, Zhejiang University of Technology, Hangzhou, Zhejiang 310014 China; 3 First Affiliated Hospital, Zhejiang University School of Medicine, Hangzhou, Zhejiang 310003 P.R. China

**Keywords:** Diabetes osteoporosis (DOP), *Epimedium brevicornum* Maxim (EP), AGE-RAGE pathway, Bioinformatics techniques

## Abstract

**Objectives:**

*Epimedium brevicornum* Maxim (EP) has a history of utilization in Chinese traditional medicine for the treatment of bone diseases. However, the precise mechanism by which EP extract (EPE) operates in Diabetes osteoporosis (DOP) remains ambiguous. The study was aimed to explore the effects and underlying mechanisms of EPE on DOP, with particular emphasis on the AGE-RAGE pathway.

**Methods:**

The DOP model was induced through a combination of a high-sugar and high-fat diet along with streptozotocin injection. Following treatment with EPE, blood glucose levels, body weight, and serum biomarkers were measured. The trabecular microstructure of the femur was analyzed using micro-CT tomography and H&E staining. Bioinformatics techniques, including network pharmacology and molecular docking, were utilized to identify key targets of EP for DOP. The predicted targets and pathways were further validated through RT-PCR, TSA analysis ELISA, and western blotting (WB), respectively.

**Results:**

The findings from animal experiments indicate that EPE has a positive impact on weight and blood glucose levels, particularly in reversing the decrease and disordered arrangement of bone trabeculae. Bioinformatics analysis reveals the involvement of the AGE-RAGE pathways in the treatment of DOP with EPE. Subsequent animal validation experiments demonstrate that EPE can regulate key proteins AGE-RAGE pathway, resulting in reducing the inflammatory factors and apoptosis, including advanced Glycation End-products (RGEs), receptor for Advanced Glycation End-products (RAGE), Interleukin-6 (IL-6), Interleukin-1β (IL-1β), Nuclear Factor Kappa B (NF-κB), BCL2-Associated X protein (Bax), B-cell lymphoma 2(Bcl2), and etc.

**Conclusion:**

This study provides clear evidence that EPE mitigates DOP through enhancement of the AGE-RAGE pathways, offering innovative insights and approaches for clinical utilization.

**Graphical abstract:**

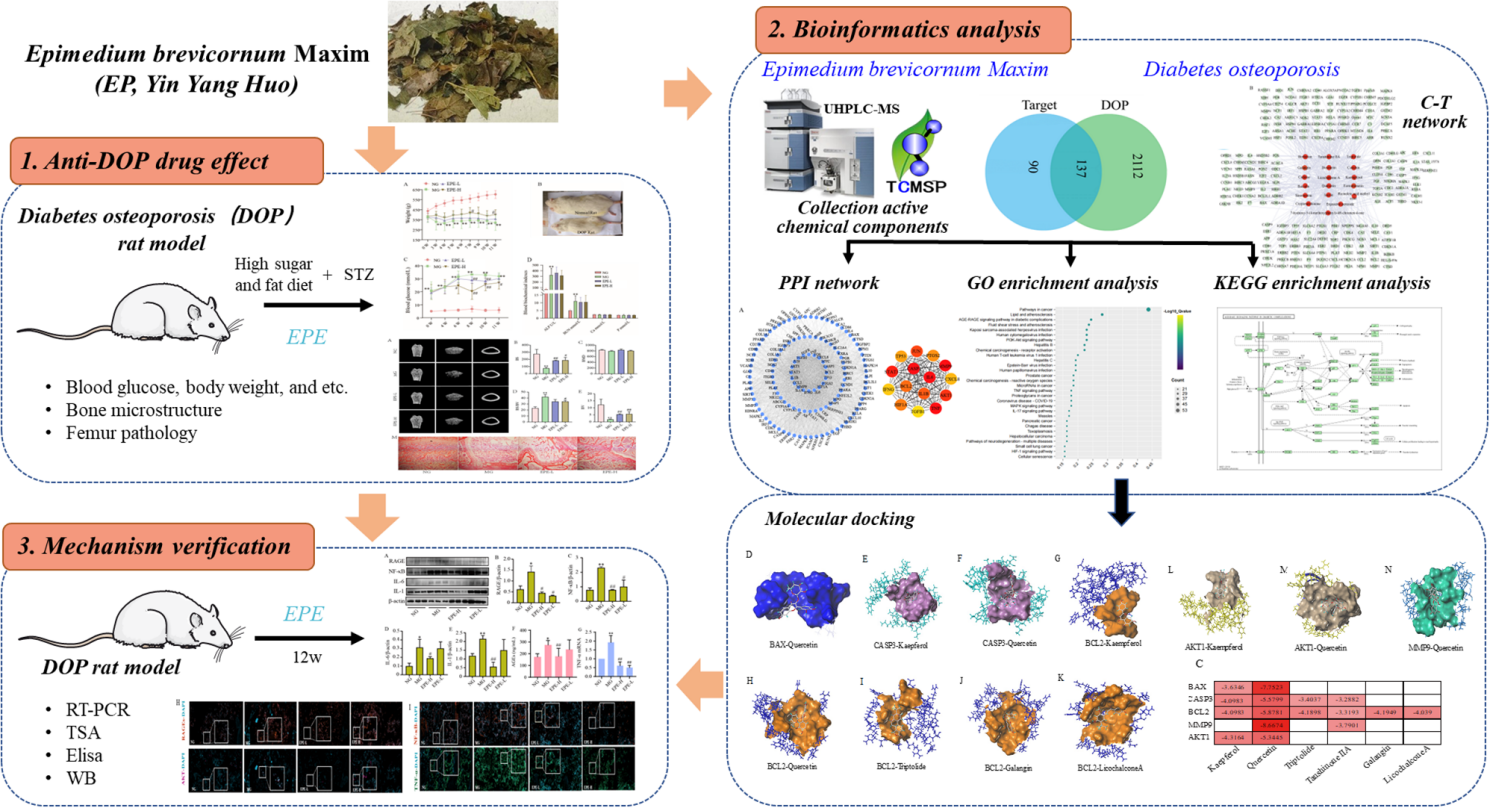

## Introduction

Diabetes osteoporosis (DOP) is a chronic musculoskeletal complication of diabetes characterized by reduced bone mass, cortical thinning, and decreased trabecular number within the bone cancellous structure (Khosla et al. 2021; Lei et al. 2023). Studies have shown that the prevalence of osteoporosis in individuals with diabetes is 4–5 times higher compared to those without diabetes (Lu et al. 2022), with a reported DOP prevalence of 37.8% in diabetic patients (Si et al. 2020). Globally, over 9 million osteoporotic fractures occur annually, with a significant proportion attributed to DOP. This poses a significant burden on healthcare systems and public health (Yang et al. 2022).

In recent years, traditional Chinese medicine (TCM) has attracted increasing attention in prevention and treatment of DOP (Lu et al. 2022). EP has been used to treat bone diseases in China for thousands of years. *Epimedium brevicornum* Maxim (EP), also known as “Yin Yang Huo,” is a widely utilized Chinese traditional medicine with a history of treating bone diseases in China for millennia (Lei et al. 2023). Contemporary research has demonstrated the anti-oxidative, anti-inflammatory, and immunomodulatory properties of EP (Xiang et al. 2017; Zhang et al. 2018). Our previous research highlighted that *Epimedii folium-Rhizoma drynariae* herbal pair effectively promoted tibia repair in the model rats (Lei et al. 2024). Further studies are warranted to explore the potential benefits of EP in the field of osteology (Xu et al. 2021). So, we want to look at the effect and mechanism of EPE on DOP.

Network pharmacology is one of the bioinformatics methods to study the material basis and mechanism of action of TCM in recent years. Based on the interaction network of “drug-target-gene-disease”, it can systematically observe the intervention and influence of drugs on disease network, and reveal the material basis and effect mechanism of the synergistic effect of TCM on the body in a holistic and systematic way (Z. WangLi 2022). The idea of network pharmacology is unified with the multi-pathway, multi-target and systematic action of traditional Chinese medicine. At the same time, the components and targets excavated by network pharmacology can be verified by molecular docking and other bioinformatics methods (Li et al. 2022). Through network pharmacology combined with molecular docking and other bioinformatics, existing studies have explored and demonstrated the advantages and characteristics of EP in the prevention and treatment of diabetic cardiomyopathy and glucocorticoid-induced osteoporosis (Liang et al. 2023).

Therefore, this study was aimed to investigate the effects and mechanisms of EPE on DOP. DOP model was established through high-sugar and high-fat diet and streptozotocin injection. Blood glucose, body weight, and serum biomarkers were detected after EPE treatment. And the trabecular microstructure and pathology of the femur were scanned by micro-CT tomography technique and evaluated with H&E staining. Then bioinformatics methods, including network pharmacology and molecular docking, were used to identify key targets of EPE for DOP, based on UPLC-MS analysis of EPE water extractment and TCMSP. And the predicted targets and pathways were verified by RT-PCR, TSA (Tyramide signal amplification) MultiMarker staining, ELISA, and western blotting (WB) respectively. This study will get novel ideas and methods for clinical applications.

## Materials and methods

### Preparation of EPE

*Epimedium brevicornum* was purchased from Zhejiang Tong Jun Tang Traditional Chinese Medicine Slices Co., Ltd. *Epimedium brevicornum* was prepared in boil water at a ratio of 1:10 for 2 h and concentrated to 10 g/mL. Then the decoction were precip

## .

itated by adding 100% ethanol to a final concentration of 70%, and the deposition washed with water and designated as EP extract (EPE). EPE solution was prepared as freeze-dried powder by Freeze dryer for 24 h.

### UPLC-MS analysis of EPE

The analysis of EPE is as described in our previous report (Lei et al. 2023), which is briefly described as follows. The EPE freeze-dried powder was diluted into 1000 µL of an 80% (*v/v*) solution of methanol, and the mixture was vortexed. The supernatant was measured by UHPLC-MS. The data was analyzed using CD2.1 software (Thermo Fisher), and the phytochemicals were identified according to the MS data and matched to mzCloud (www.mzcloud.org).

### Preparation of type 2 DOP rat model

8-week-old male rat were purchased from Shanghai Shrek laboratory animal Co., Ltd (Shanghai, China). The rats used in the present study were treated in accordance with the Guide for the Care and Use of Laboratory Animals. All animal experiments were performed with the approval of the Ethics Committee of Zhejiang Academy of Traditional Chinese Medicine.

After 30 days adaptive feeding, rats were fed with high sugar high-fat diet (Beijing Boaigang biological technology co., Ltd, 1135D) for 4 weeks. Then all rats were fasted for 12 h. The 1.0% STZ solution dissolved with citric acid buffer was injected once at the weight of 35 mg/kg by ip. Blood glucose was measured with tail vein on the 3th, 5th, and 7th days after modeling. Diabetes mellitus model was successfully modeled when the fasted blood glucose was more than 11.0mmol/L. Finally, there were twenty-four DOP rats and eight normal rats were used for further experiment.

### Determination of blood glucose, body weight, and serum biomarkers

The number of twenty-four DOP rats were randomly divided into three groups according to blood glucose and body weight, following as EPE freeze-dried powder 100 mg/kg group (EPE-L), EPE freeze-dried powder 200 mg/kg group (EPE-H), and DOP model group (MG). Another number of eight normal rats were set as normal group (NG). The EPE group rats were intraperitoneally administrated with 100 and 200 mg/kg/day daily for 12 weeks, respectively. The MG and NG were administrated with purified water under the same conditions. Body weights were detected every one weeks and blood glucose was measured with tail vein blood every two weeks after administered. At the end of experiment, rats were anesthetized with pentobarbital sodium and blood was taken for determination of ALP, BUN, P, and Ca^2+^ by an automatic biochemical analyzer.

### Analysis of bone microstructure

At the end of experiment, the trabecular microstructure of the femur was scanned by micro-CT tomography technique (SkyScan 1176, Institute of Hydrobiology, Chinese Academy of Sciences) with a resolution of 18 μm. The scanned images from each group were evaluated at the same thresholds and made three-dimensional structural reconstruction of each sample. Air borne software CT analysis was used to detect trabecular microstructure parameters are as follows: bone volume (BV), bone volume/totalvolume (BV/TV), trabecular bone number (Tb.N), trabecular spacing (Tb.Sp), trabecular thickness (Tb.Th), structure model index (SMI), Bone Surface (BS), and Conn-Dens. Bone histopathology analysis by H&E staining of the femur, carried out according to our previous studies (Lei et al. 2021).

### Evaluation of the femur pathology

At the end of experiment, the pathology of the femur was evaluated with H&E staining. As the previous study (Hu et al. 2023), the femur were fixed dehydrated, embedded, and sectioned. Then all of the specimens were sliced for H&E staining according to the kit instructions.

### Network Pharmacology analysis and preliminary verification of molecular docking

#### Collection and screening of active chemical components

Combined with UHPLC-MS identification of EPE, mainly relying on the Traditional Chinese Medicine System Pharmacology Database and Analysis Platform (TCMSP) created by the College of Life Sciences, Northwest A&F University, to query and summarize the chemical components of EPE. Then, through the ADME module in the TCMSP database, set the parameters OB ≥ 30% and DL ≥ 0.18 to screen and collect potential active ingredients of compounds.

#### Target protein screening and network construction

By using the Drug bank database to identify drug molecules with known biological activity and target annotation information, a three-dimensional molecular similarity method is applied to predict potential targets for query molecules. At the same time, Genecards was used to search for target genes related to diabetes bone hyperplasia. Integrating the screening results of the two, we can further obtain the targets of diabetes bone hyperplasia corresponding to different active chemical components, so as to establish the correspondence between the active components of EPE and target proteins, and build a compound target network.

The Cytoscape 3.3 software is used to construct an interaction network between traditional Chinese medicine, components, and targets. The medicinal materials, components, and targets are represented by nodes, and the interactions between nodes are represented by edges. Then, the Network Aalyzer plugin is used to analyze the degree of network topology properties. The higher the degree, the more important the compound or target is, and it plays a key role in treating diseases.

#### Protein protein interaction (PPI) and hub gene analysis

After knowing the target proteins corresponding to the active chemical components, use the Uniprot database(http://www.uniprot.org/)Find the gene name corresponding to the target protein. Import the obtained target proteins into the String database for protein-protein interaction analysis. In the STRING online database, the creature was selected as Homo sapiens, with a minimum interaction requirement score of 0.9. Meanwhile, import the PPI data obtained into cytoscape for visual analysis, and use the cytoHubba plug-in to find the key genes that map EPE to the pathogenesis of DOP.

#### GO enrichment analysis and KEGG pathway analysis

As described in our previous research (Lei et al. 2023), enrichment of the Gene Ontology (GO) term was analyzed using DAVID (https://david.ncifcrf.gov), and the KEGG pathway was analyzed using the KEGG Orthology-Based Annotation System (KOBAS) database (http://kobas.cbi.pku.edu.cn/). The visualization of GO terms and KEGG pathway were generated with ChiPlot (https://www.chiplot.online/). The entries with corrected p-values less than 0.05 were retained and the top 10 was further analyzed.

#### Preliminary verification of molecular Docking

Based on the results of network pharmacology and literature research, molecular docking validation was conducted on the main components and pathway targets predicted by network pharmacology. From PDB database (https://www.rcsb.org/) obtain the 3D structure of the target protein and obtain the corresponding 3D structure of the active ingredient from the TCMSP database using Auto dock software (https://autodock.scripps.edu/). Perform molecular docking with a semi flexible docking mode, select genetic algorithm as the retrieval parameter, set the docking frequency to 50 times, and select the binding mode with the lowest binding energy as the optimal result for ligand receptor binding.

We get the 3D structure of protein from PDB database (https://www.rcsb.org/) and obtain corresponding 2D structure of the active ingredient from the NCBI database (https://www.ncbi.nlm.nih.gov/). Than we use SYBYL software to dock molecular structure with docking mode-Surflex-Dock Geom and the docking number was 20.

### Real-time polymerase chain reactions (RT-PCR)

The femurs were crushed on an ice platform and a bone tissue mRNA extraction kit (Aidlab, Beijing, China) was used to extract total RNA from bone tissue. The expression levels of *Akt*,* Fas*,* Fasl*,* TNFα*, *Runx2*, *Bax*, and *Bcl2* were measured as previously described (Lei et al. 2023).

### TSA (Tyramide signal amplification) multimarker staining technique

The protein expression of NF-κB, TNF-α, RAGEs, and AKT were detected by TSA multimarker staining technique in bone tissues. The test kit was purchased from Haoke Biotechnology Co. Ltd (HKI000-5 A) and operation was measured according to the manufacturers’ instructions. In short, 4 μm paraffin slices were dewaxed with xylene and rehydrated with gradient ethanol. Slices were washed three times with PBS. The slices were blockaded by 0.3% hydrogen peroxide and antigen repair by citrate buffer. Then the slices were blockaded with 5% BSA for 30 min, and primary antibodies (1:200) were added and incubated overnight at 4 ◦C. After washing, 150 µL HRP secondary antibody was added and incubated for 1 h at 37 ◦C. The slices were then stained with different color fluorescent dyes. The antibody clearance solution was washed and incubated with another primary antibody. After all antibodies were incubated, the slides were mounted with DAPI-containing medium to counters tainnuclei and then observed under a florescent microscope.

### Western blot analysis

The detection of IL-6, IL-1β, NF-κB, MMP9, ALP and RAGE expression via western blot analysis was conducted in accordance with the methodology outlined in a previous study (Lei et al. 2023). In summary, femurs were crushed on an ice platform and total proteins were extracted using lysis buffer. Equal amounts of protein (20 µg) were separated by 12% SDS-PAGE and transferred to a 0.45 μm PVDF membrane. Following blocking with 5% BSA at 37 °C for 1 h, the membranes were incubated with homologous primary antibodies diluted at 1:1000 in PBST at 4˚C overnight, followed by washing. The washed membrane was further labeled with HRP-conjugated secondary antibody (1:5000 dilution) for 2 h at room temperature. The blotted protein bands were detected by chemiluminescent assay kit (Bio-rad, America) and the protein expression levels were normalized to β-actin.

### ELISA method to detecte the ages level in serum

AGEs (Bioswamp, RA20685) in serum samples were measured according to the manufacturers’ instructions with the ELISA kit.

### Statistical analysis

Data was reported as mean ± SD and analyzed using SPSS 16.0 software. Student’s t-tests or ANOVA were used for group comparisons, with *p* < 0.05 considered significant.

## Results

### Active ingredients in EPE

**Fig. 1:**
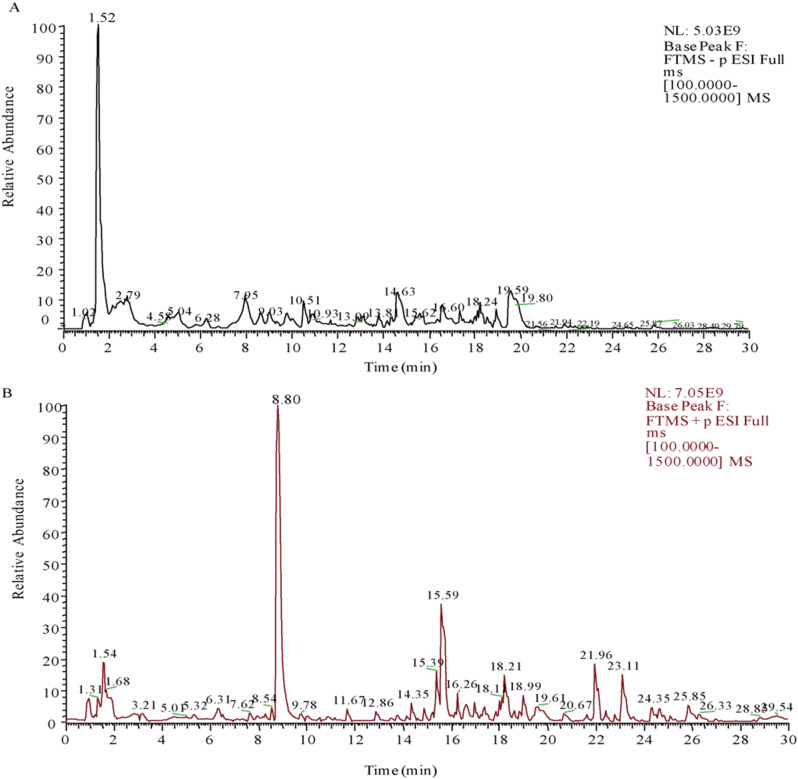
Characterization of EPE by LC-MS. (**A**) Negative ion model diagram. (**B**) Positive ion model diagram

By UHPLC-MS on EPE, the total ion flow diagrams, with a total of 829 compounds identified, were shown in Fig. 1. The 18 compounds with the highest relative abundance from the UHPLC chromatogram are shown in Table 1. Among them, Epimedin C, Icaritin, citric acid, and Epimedin B, which are known bioactive components of EPE, were detected.

**Table 1:** Characterization of EPE by LC-MS

Name	Formula	RT min	Ration (%)
6-Acetylcodeine	C_20_H_23_NO_4_	8.808	15.44
D-(-)-Quinic acid	C_7_H_12_O_6_	1.499	9.37
Icariin	C_33_H_40_ O_15_	15.616	5.38
Citric acid	C_6_H_8_O_7_	2.781	4.48
DL-Malic acid	C_4_H_6_O_5_	1.619	3.09
(6 S)-N-(4-Chlorobenzyl)-5-{[5-(hydroxymethyl)-2-furyl]methyl}-4,5,6,7-tetrahydro-1 H-imidazo[4,5-c]pyridine-6-carboxamide	C_20_H_21_ClN_4_O_3_	14.634	2.17
Icaritin	C_21_H_20_O_6_	18.232	1.95
1-Stearoylglycerol	C_21_H_42_O_4_	23.123	1.85
Epimedin B	C_38_H_48_O_19_	15.413	1.47
Taurochenodeoxycholic Acid (sodium salt)	C_26_H_45_NO_6_S	16.434	1.13
Epimedin C	C_39_H_50_O_19_	15.695	1.12
Betaine	C_5_H_11_NO_2_	1.5	1.06
D-(+)-Pyroglutamic Acid	C_5_H_7_NO_3_	2.448	0.95
1,2,3-cyclopropanetricarboxylic acid	C_6_H_6_O_6_	4.493	0.89
Oxymatrine	C_15_H_24_N_2_O_2_	6.331	0.68
DL-Stachydrine	C_7_H_13_NO_2_	1.539	0.60
13(S)-HOTrE	C_18_H_30_O_3_	18.967	0.58
3-(2,4-dihydroxyphenyl)-7-hydroxy-6,8-bis(3-methylbut-2-en-1-yl)-4 H-chromen-4-one	C_25_H_26_O_5_	20.714	0.58

### Effect of EPE on improving weight, blood glucose, and serum biochemical indicators

**Fig. 2:**
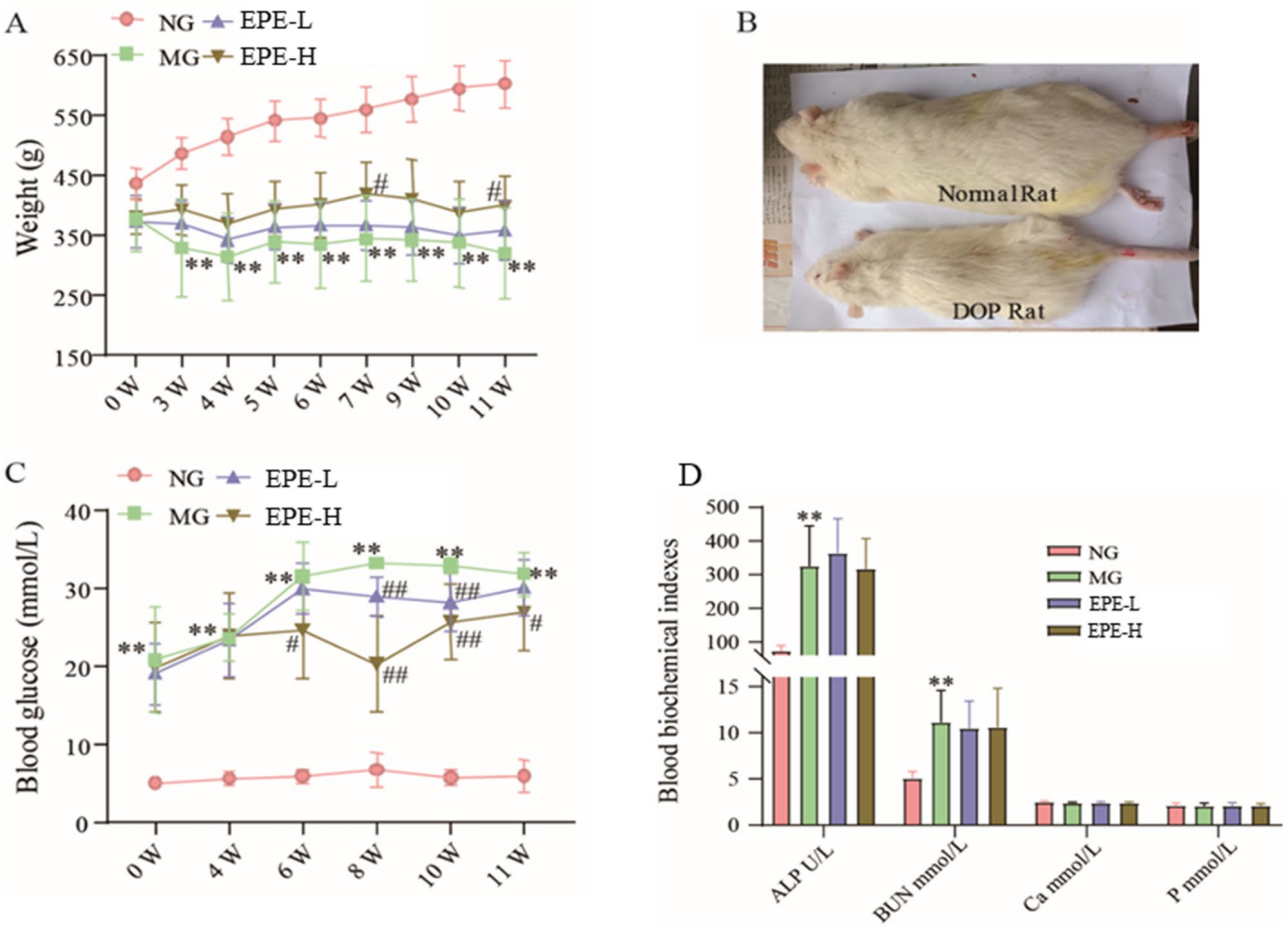
Effect of EPE on improving body weight, blood glucose, and serum biochemical indicators. (**A**) Body weight during treatment for 11 weeks. (**B**) Morphology between normal rat and DOP rat staining. (**C**) Blood glucose. (**D**) Serum biochemical indicators. The data were presented as the means ± SD. **p < 0.01, vs. normal group (NG), ^#^*p* < 0.05, ^##^*p* < 0.01 vs. model group (MG)

As shown in Fig. 2A and B, DOP rats were showed a significantly decreased body weight during the experimental period compared to the normal group, and EPE 200 mg/kg treatment suppressed the decrease in the body weight compared to the model group after 7 and 11 weeks treatment, respectively (*p* < 0.05). Moreover, DOP rats were showed a significantly increased of blood glucose during the experimental period compared to the normal group, and EPE 200 mg/kg and 100 mg/kg could decrease the blood glucose level after treatment for 6 and 8 weeks, respectively (*p* < 0.05, 0.01) (Fig. 2C).

Blood biochemical indicators of ALP and BUN were significantly increased in the model group, but EPE had no effect on them (Fig. 2D). There was no difference in the serum Ca^2+^ and P concentration of rats in all groups (Fig. 2D). These results indicated that EPE could change the body weight and blood glucose level of the model rats, which was beneficial to the prevention and treatment of diabetes osteoporosis.

### Effect of EPE on improving bone microstructure and pathology

**Fig. 3:**
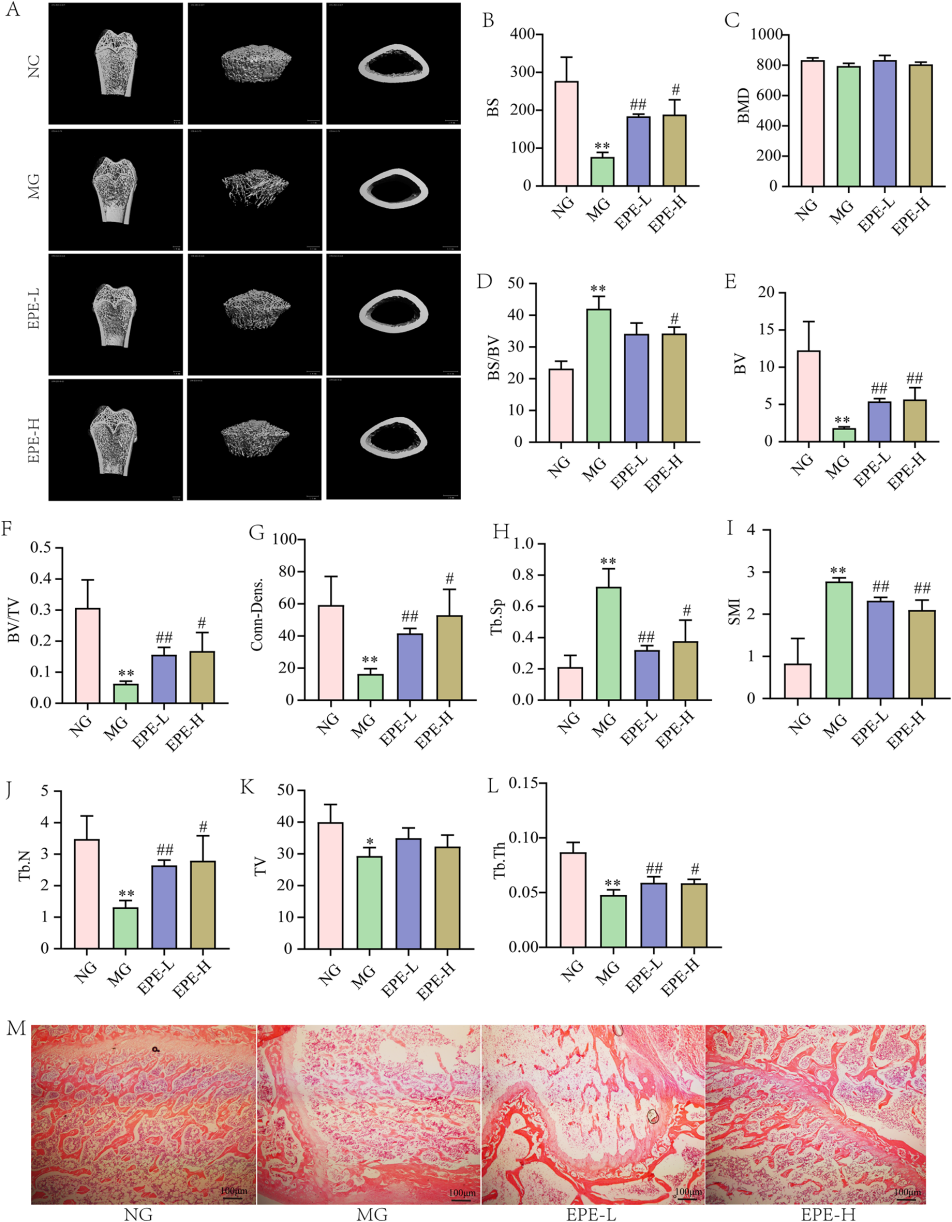
EPE accelerated bone consolidation in diabetic rats. (**A**) Representative 3Dimages of thefemur. (**B**)~(**J**) Quantitative analysis of bone microstructure, as follows bone mineral density (BMD), bonevolume (BV), bonevolume/tissue volume (BV/TV), trabecular number (Tb.N), degree of trabecular separation (Tb.Sp), trabecular thickness (Tb.Th), structure model index (SMI), bone surface (BS), connection density (Conn-Dens). (**M**) Representative photomicrograph of histopathology observation of the femur with H&E staining (100x). The data were presented as the means ± SD. ^*^p < 0.05, ^**^p < 0.01, vs. normal group (NG), ^#^*p* < 0.05, ^##^*p* < 0.01 vs. model group (MG)

It is well known that diabetes status could break the balance of bone remodeling. micro-CT was used to observe the microstructure of the femur, with BV, BV/TV, Tb.N, Tb.Sp, Tb.Th, SMI, BS, and Conn-Dens analyzed (Fig. 3). Compared to the normal group, the BV, BV/TV, Tb.N, Tb.Th, BS, and Conn-Dens of DOP rats significantly decreased (*p* < 0.01), and Tb.Sp and SMI obviously increased (*p* < 0.01), indicated that the diabetic model rats showed bone hyperplasia. Fortunately, EPE 200 mg/kg and 100 mg/kg could reverse these bone hyperplasia changes (*p* < 0.05, 0.01) (Fig. 3). These results suggested that EPE could ameliorate the diabetes osteoporosis.

Further, we detected the bone pathology of diabetes rats after 11 weeks administrated with EPE. The H&E result of the femur was shown in Fig. 3M. Compared to the normal group, the bone trabeculae of DOP rats were thin, disorderly arranged and structure imperfect. At the same time, the number of bone trabeculae was significantly reduced. The model rata was obvious osteoporosis statue. Compared to the model group, EPE reversed the phenomenon of bone trabeculae decreasing and disorderly arranged.

### Network Pharmacology analysis

#### Construction of active component-target (C-T) network

We put all UHPLC-MS identification of EPE and combined with TCMSP database (http://lsp.nwu.edu.cn/tcmsp.php) of traditional Chinese medicine compounds in search of EP, received 123 kinds of chemical composition. Further screening by OB ≥ 30% and DL ≥ 0.18, 20 compounds with good ADME properties were obtained for follow-up studies, as shown in Table 2.

**Fig. 4:**
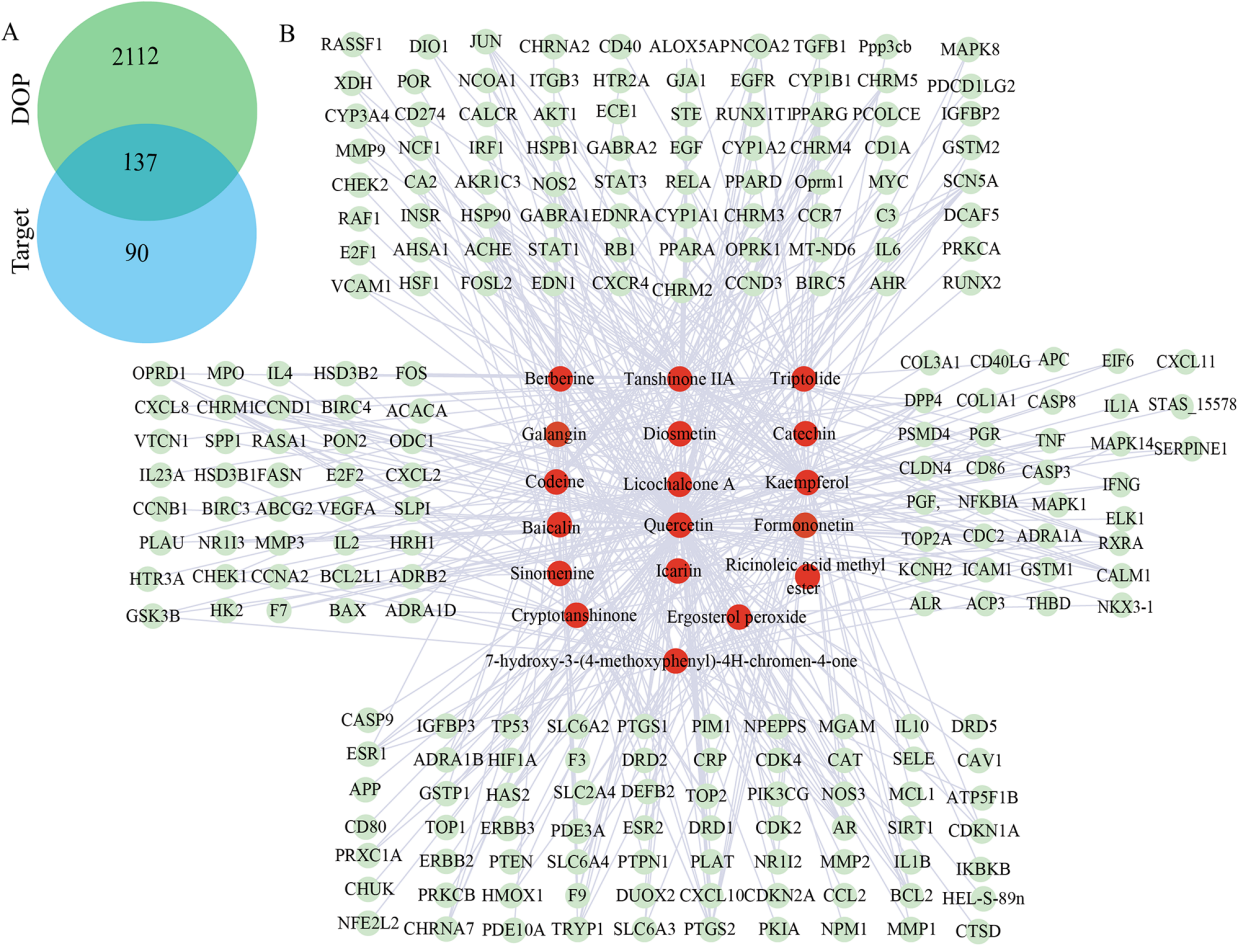
EPE active component-target (C-T) network. (**A**) The Venn diagram illustrates the intersection of target genes in EPE and DOP. (**B**) Component-target network of the intersection of target genes in EPE and DOP (The red circles represent the active ingredients, the green circles represent the targets, and the connecting lines indicate the correlation between targets and active compounds)

We construct the target network diagram corresponding to diabetic osteoporosis disease, further screen the target of active ingredients and predict the target related to diabetic osteoporosis disease, and eliminate the compounds with no corresponding target and no corresponding human target gene in Uniprot. Finally, we obtain 225 targets corresponding to 18 compounds. DOP disease genes associated with disease 4497 were found in GeneCards database, selecting correlation median score above 2249. Then we use the website (https://jvenn.toulouse.inrae.fr/app/example.html) to change the corresponding target compounds associated with diabetic osteoporosis disease targets in intersection, received a total of 137 target genes, as shown in Fig. 4A. The active ingredient-disease target network (C-P network) was further constructed. C-P network has 243 nodes, forming 498 relational pairs. Most active compounds can interact with multiple target proteins, as shown in Fig. 4B.

**Table 2:** Main active substance parameters of EPE

Active ingredient	OB (%)	DL
Ricinoleic acid methyl ester	36.3	0.19
Galangin	45.55	0.21
Formononetin	69.67	0.21
7-hydroxy-3-(4-methoxyphenyl)-4 H-chromen-4-one	69.67	0.21
Kaempferol	41.88	0.24
Catechin	54.83	0.24
Quercetin	46.43	0.28
Licochalcone A	40.79	0.29
Monoolein	34.13	0.3
Docosatrienoic acid	43.23	0.3
Cryptotanshinone	52.34	0.4
Sinomenine	30.98	0.46
Codeine	45.48	0.56
Diisodecyl phthalate	41.08	0.57
Icariin	41.58	0.61
Triptolide	51.29	0.68
Baicalin	40.12	0.75
Berberine	36.86	0.78
Ergosterol peroxide	40.36	0.81
Diosmetin	31.34	20.27

#### PPI network and molecular Docking

**Fig. 5:**
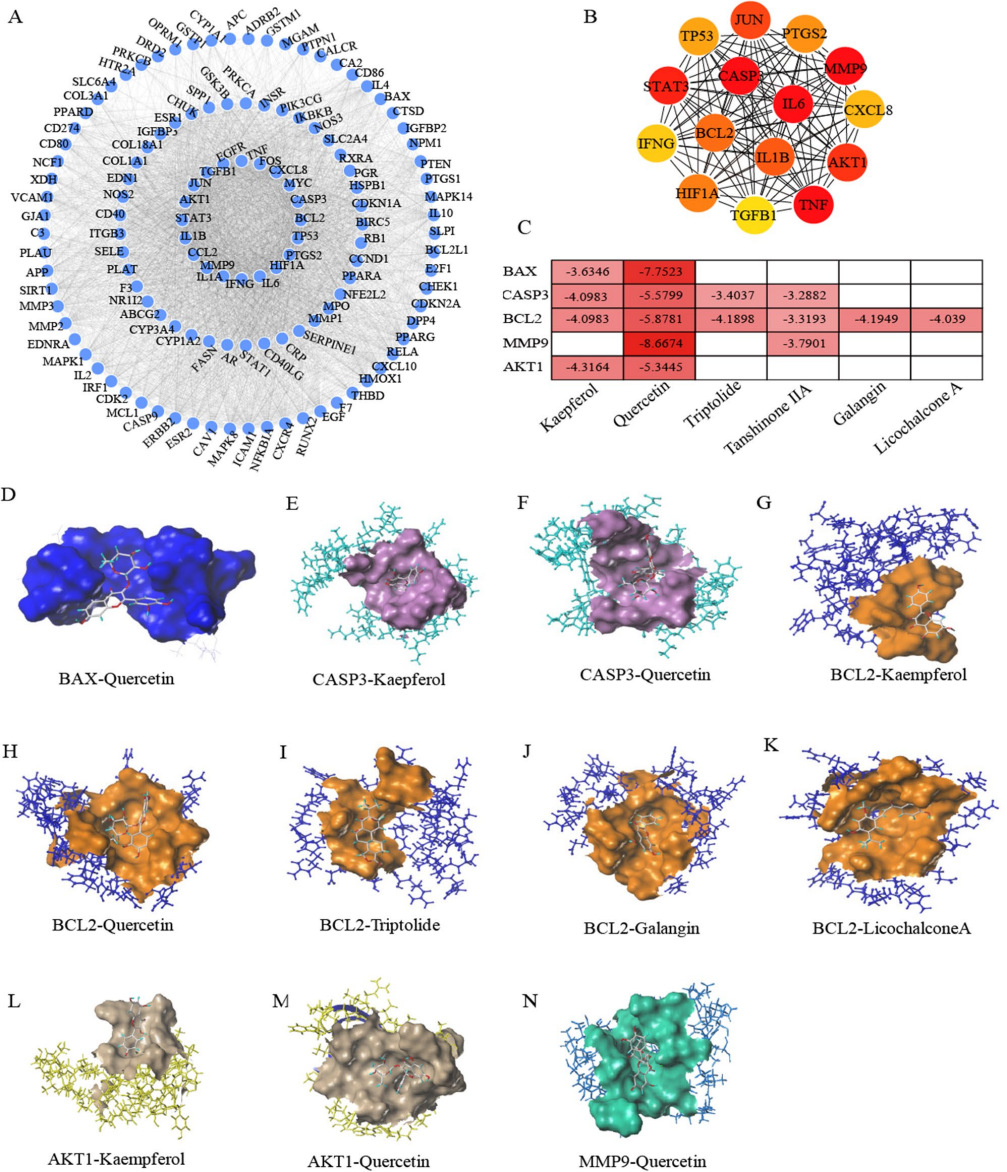
PPI network and molecular docking. (**A**) PPI network of the 129 complementary target proteins. (**B**) The top 15 hub targets based on MCC algorithm. (**C**) The heat map present molecular docking results between hub targets and active compounds. (**D** ~ **N**) Docking score is greater than or equal to 4.0 kcal/mol between active compounds and targets

To construct PPI network, then analyze and predict hub target based on MCC algorithm. Protein interaction analysis was carried out on the selected targets. A total of 3387 protein interaction relationships were formed on 129 targets. We imported the protein interaction network into Cytospase software and analyze by cytoHubba plug-in to obtain the top 15 key genes, as shown in Fig. 5A ~ B. Analysis of these key genes showed that most of them were involved in apoptosis, cell cycle and cell proliferation, such as CASP3, MMP9, BCL2, IL6, TNF, IL1B, IL1A, CCL2 and so on are mainly involved in inflammation. These results suggested that diabetic osteoporosis may have therapeutic effects by influencing the biological processes involved in these key target genes.

We identified potential pathways of action and validated molecular docking based on network pharmacology and literature findings. We selected BAX, BCL2, and CASP3 as target proteins. The docking score is mainly related to binding crash and intermolecular polarity. The lower the binding energy is, the more facile the docking. A score of more than 4 indicates that the ligand has good binding activity to the target. The docking results the combination of Quercetin with BAX and MMP9 were the most significant. We make heat map and best results of molecular docking are shown in the Fig. 5C ~ N.

#### The GO and KEGG enrichment analysis

**Fig. 6:**
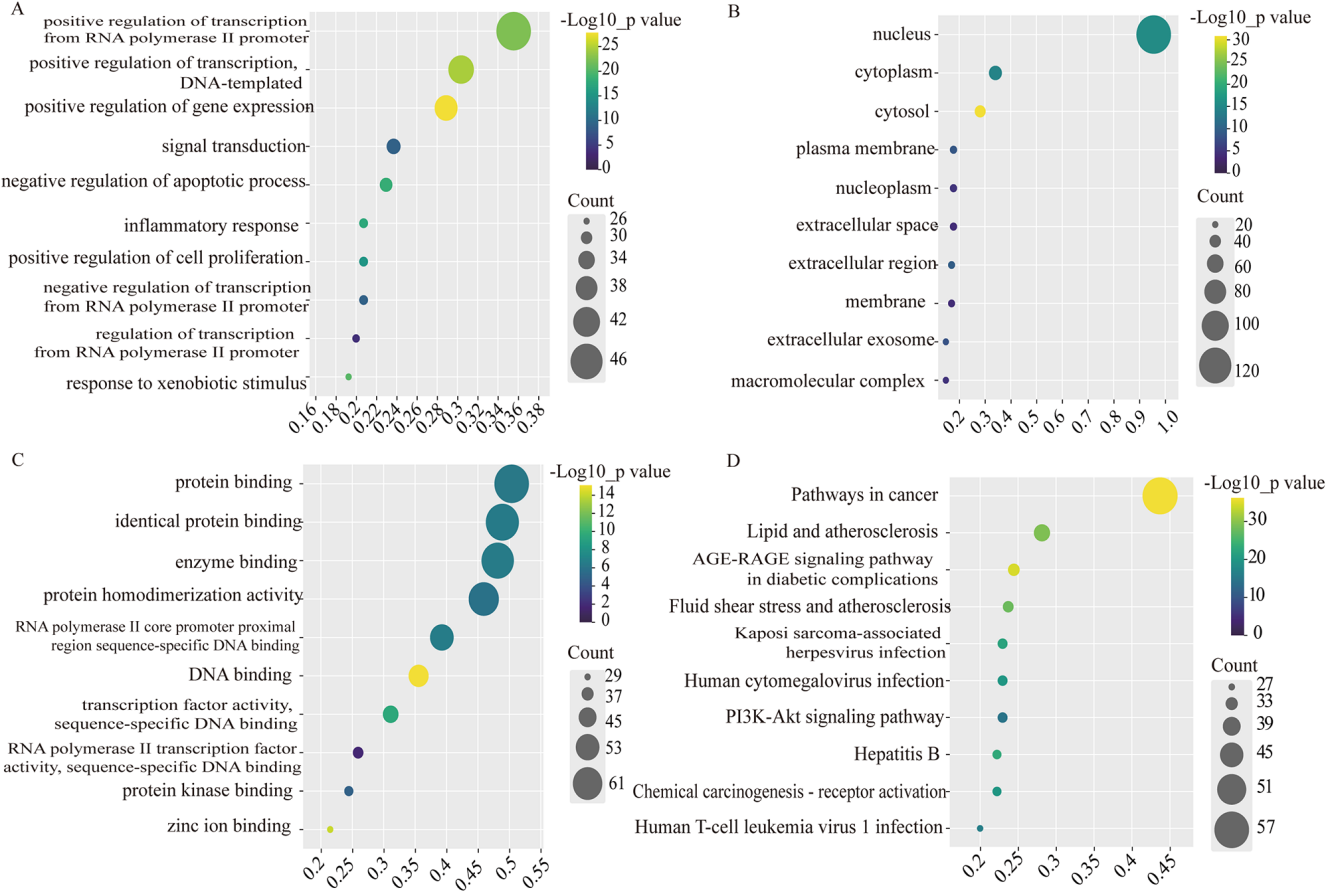
GO and KEGG analysis. (**A**) The top 10 terms of BP; (**B**) The top 10 terms of the CC; (**D**) The top 10 terms of MF; (**D**) The top 10 terms of KEGG pathway. The dot was larger, the more the number of genes. BP was biological process; CC was cellular component; MF was molecular function

**Fig. 7:**
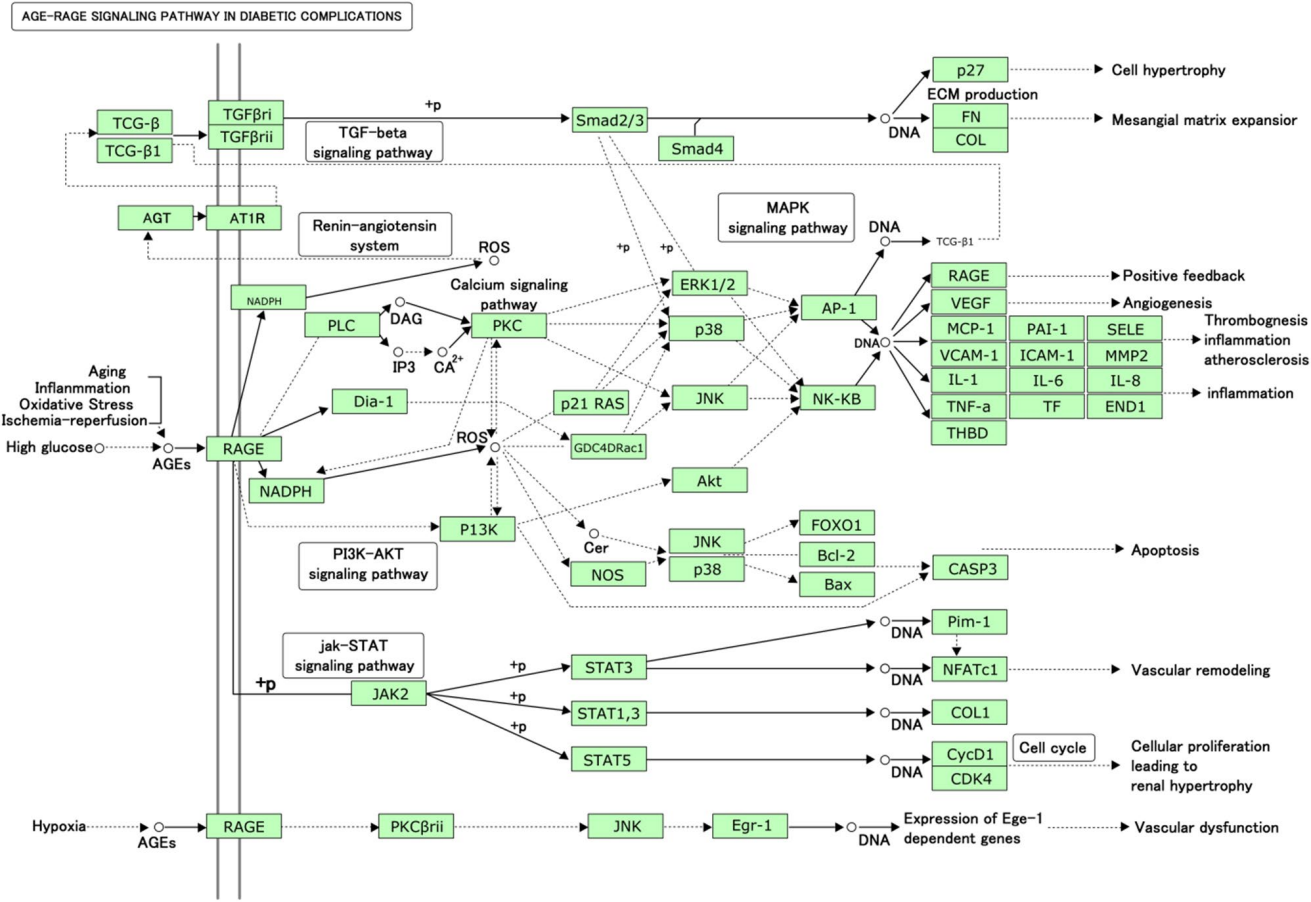
AGE-RAGE signaling pathway in diabetic complications (hsa04933)

The top 10 terms for sub-ontology were shown in Fig. 6A ~ C. The BPs mainly involved positive regulation of transcription from RNA polymerase II promoter, inflammatory response and so on. The MFs mainly involved protein binding. The CCs mainly involved nucleus. The KEGG analysis was shown in Fig. 6D. The targets were primarily linked with multiple signaling pathways, such as the AGE-RAGE signaling pathway, PI3K-Akt signaling pathway, and so on. One of them that caught our attention was the AGE-RAGE signaling pathway in diabetic complications of DOP (see Fig. 7).

### EPE improved bone reconstruction in the DOP rat

**Fig. 8:**
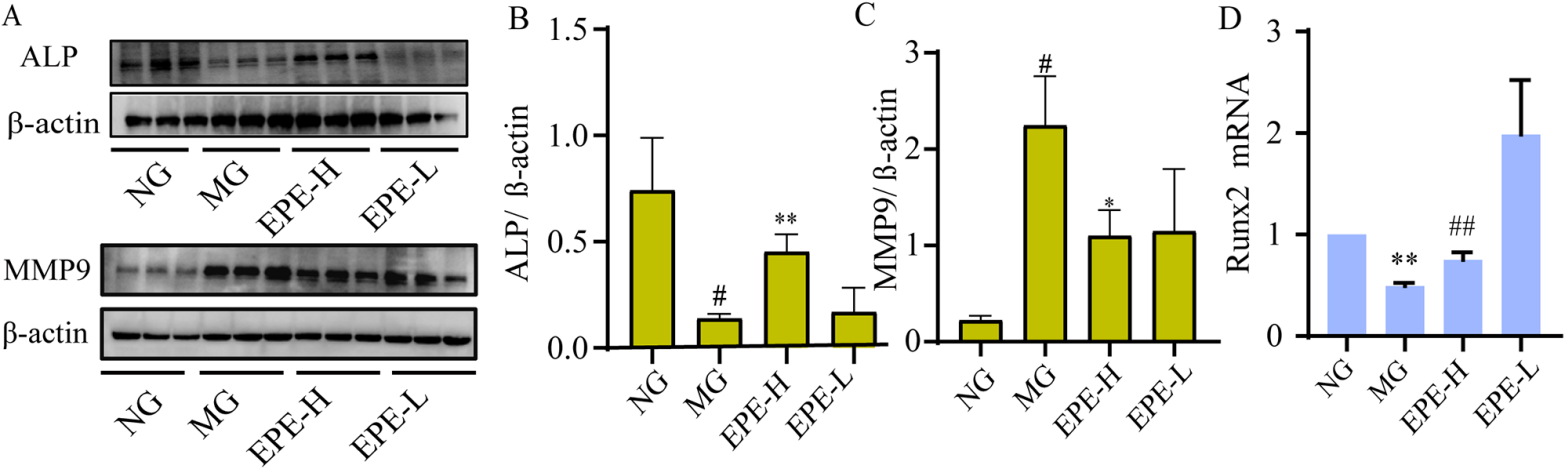
EPE improved bone reconstruction in the DOP rat femur. (**A**) Representative blot expression of ALP and MMP in the femur. (**B**) Semi-quantitative analysis ALP expression. (**C**) Semi-quantitative analysis MMP expression. (**D**) *Runx2* mRNA expression. Data are presented as the mean ± SD, *p < 0.05, **p < 0.01, vs. normal group (NG), ^#^*p* < 0.05, ^##^*p* < 0.01 vs. model group (MG)

RUNX2 and ALP are important markers of osteogenic differentiation. MMP9 is closely related to osteoporosis or osteoclasts, and MMP9 is also one of the important targets for our network pharmacological prediction. Western blot and RT-PCR were used to detect the protein expressions of ALP and MMP9, and the mRNA expression of *RUNX2*, respectively. The results shown that in the model control group, the protein expression of ALP and the genes expression of *RUNX2* were significantly down-regulated (*p* < 0.01, 0.05), and the protein expression of MMP9 was down-regulated compared to control group (Fig. 8). After treatment with EPE for 12 weeks, the expression of ALP and *RUNX2* were remarkable elevated in EPE-H group (*p* < 0.01, 0.05), and the expression of MMP9 was down-regulated (*p* < 0.05) (Fig. 8C). These results suggested that EPE could improve bone reconstruction by regulating ALP, RUNX2, and MMP9 expression.

### EPE alleviated bone reconstruction by inhibiting apoptosis in the DOP rat

**Fig. 9:**
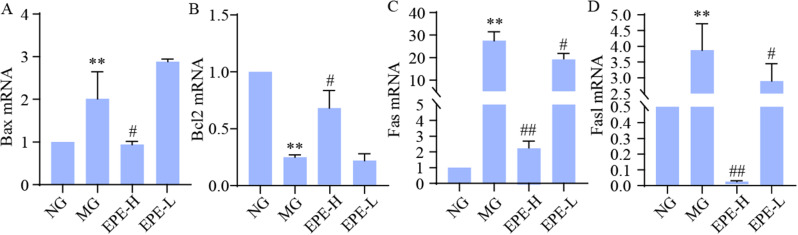
Effects of EPE on the hub targets mRNA expression and AGEs level. (**A**) *Bax* mRNA expression. (**B**) *Bcl2* mRNA expression. (**C**) *Fas* mRNA expression. (**D**) *Fasl* mRNA expression. Data are expressed as the mean ± SD. *p < 0.05, **p < 0.01, vs. normal group (NG), ^#^*p* < 0.05, ^##^*p* < 0.01 vs. model group (MG)

Apoptosis can inhibit bone reconstruction. We further focused on using RT-PCR to detect the mRNA levels of bone reconstruction and apoptosis related indicators, including *Bax*,* Fas*,* Fasl*, and *Bcl2.* And BAX and BCL2 are also important targets for network pharmacological prediction. The results showed that in the model control group, the genes expression of *Bax*, *Fas*, and *Fasl* were significantly up-regulated (*p* < 0.01), the expression of *Bcl2* were significantly down-regulated (*p* < 0.01) compared to control group (Fig. 9). After interred with EPE-H for 12 weeks, the expression of *Bax*, *Fas*,* and Fasl* were remarkable depressed (*p* < 0.01, 0.05), and the genes expression of *Bcl2* were significantly increased (*p* < 0.01, 0.05). Moreover, EPE-L administrated also down-regulated the genes of *Fasl* expression (*p* < 0.01, 0.05).

### EPE alleviated bone reconstruction by regulating AGE/RAGE pathway in the DOP rat

**Fig. 10:**
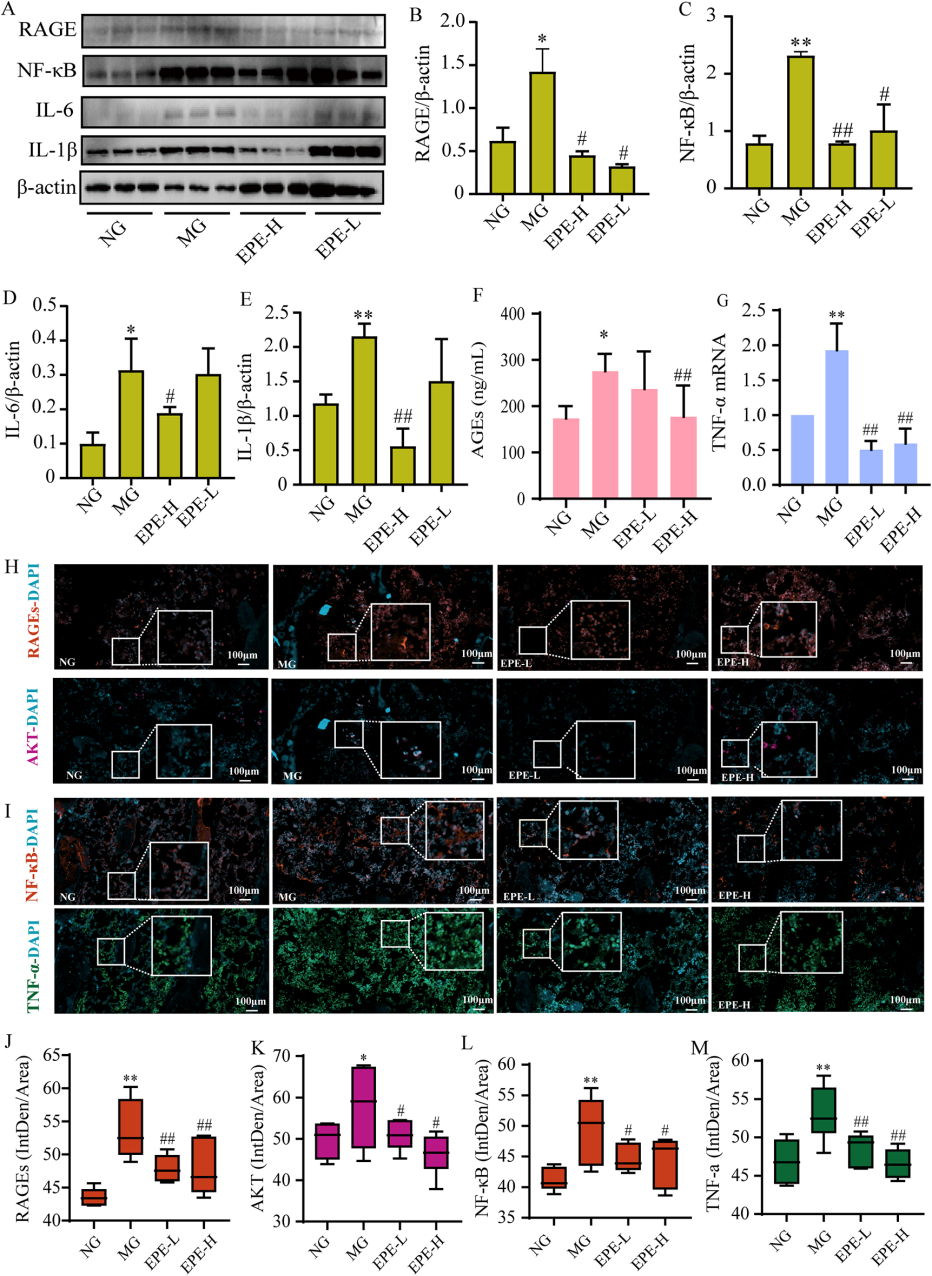
EPE reduced inflammatory by AGEs/RAGE/NF-κB pathway in the DOP rat femur. (**A**) Representative blot expression of RAGE, NF-κB, IL-1β, and IL-6 in the femur. (**B**) Semi-quantitative analysis RAGE expression. (**C**) Semi-quantitative analysis NF-κB expression. (**D**) Semi-quantitative analysis IL-6 expression. (**E**) Semi-quantitative analysis IL-1βexpression. (**F**) AGEs level in serum. (**G**) TNF-α mRNA expression in the femur. (**H**) Representative blot expression of ALP in the femur. (**A**) Representative expression of RAGEs/AKT in the femur. (**B**) Representative expression of NF-κB/TNF-α in the femur. (**C**) Semi-quantitative analysis RAGEs expression. (**D**) Semi-quantitative analysis AKT expression. (**E**) Semi-quantitative analysis NF-κB expression. (**F**) Semi-quantitative analysis TNF-α expression. Data are presented as the mean ± SD, *p < 0.05, **p < 0.01, vs. normal group (NG), ^#^*p* < 0.01, ^##^*p* < 0.01 vs. model group (MG)

In our research we detected the serum level of AGEs by Elisa assay. The results shown that AGEs were significantly increase in model group (*p* < 0.05) and EPE-L administrated could significant reduced the contents compared to model group (*p* < 0.01) (Fig. 10). Western blot was further used to detect AGE-RAGE signaling pathway relative protein expression. We got similar results. In the model control group, the protein expression of IL-6, IL-1β, NF-κB, and RAGE were significantly up-regulated (*p* < 0.01, 0.05) compared to control group (Fig. 10A ~ E). After interred with EPE for 12 weeks, the expression of IL-6, IL-1β, NF-κB, and RAGE were remarkable depressed in EPE-H group (*p* < 0.01, 0.05), and similar results were observed for NF-κB, and RAGE in EPE-L group (Fig. 10A ~ E).

In the model control group, the protein expression of RAGEs and AKT were significantly up-regulated (*p* < 0.01) compared to control group (Fig. 10H, J, K). After interred with EPE for 12 weeks, the expression of RAGEs and AKT were remarkable depressed (*p* < 0.01, 0.05). Further the protein expression of TNF-α and NF-κB were significantly up-regulated (*p* < 0.01) in the model control group compared to control group (Fig. 10I, L, M). And EPE could reverse the changes of these indicators (*p* < 0.01, 0.05). These results suggested that EPE could alleviate AGE-RAGE signaling pathway relative genes expression and reduce AGEs contents.

## Discussion

Diabetic osteoporosis (DOP) refers to a systemic metabolic bone disease in diabetic patients, which is caused by increased blood glucose and metabolic changes in the body, resulting in decreased bone mineral density, damaged bone microstructure, increased bone fragility, and easy to fracture (Khosla et al. 2021; Lu et al. 2022). DOP has a high incidence in especial the prevalence of DOP in diabetic patients is 37.8% (Si et al. 2020). Therefore, the study on the pathogenesis and drug therapy of DOP is of great significance.

An imbalance in bone homeostasis is well known to be caused by more osteoclast-mediated bone resorption than osteoblast-mediated bone formation results in osteoporosis. It has been reported that the fracture of cancellous bone and the mass and quantity of bone trabeculae significantly decreased in DOP model rats. EP has been used to treat bone diseases in China for thousands of years. Our previous research highlighted that the polysaccharide isolated from EP has positive effects on cell growth and protective effects on cellular injury (Lei et al. 2023). This study adopts classical DOP model, established through high-sugar and high-fat diet and streptozotocin injection (Rao et al. 2021), to investigate the effects and mechanisms of EPE on DOP rat. Our results shown that DOP rats were showed a significantly increased of blood glucose with changes in bone microstructure, including the BV, BV/TV, Tb.N, Tb.Th, BS, and Conn-Dens decreased, and Tb.Sp and SMI obviously increased. Fortunately, EPE could reverse these bone hyperplasia changes.

Traditional Chinese medicine (TCM) has multi-component and multi-target effects. Network pharmacology is an important means to explain the mechanism of action of TCM in treating diseases (Z. WangLi 2022; Li et al. 2022). At the same time, the components and targets excavated by network pharmacology can be preliminatively verified by molecular docking and other bioinformatics methods (Li et al. 2022). Based on UPLC-MS analysis of EPE and TCMSP, screening by ADME (OB ≥ 30% and DL ≥ 0.18), 20 compounds in EPE with good properties were obtained quercetin, triptolide, kaepferol, icariin, and galangin may have anti-DOP effects. It had been reported that quercetin, triptolide, and galangin were effective in treating osteoporosis (Cui et al. 2020; Deng et al. 2024; Li et al. 2023), and Quercetin also has a clear effect on improving bone microstructure in DOP mice (BaşAlbeniz 2022).

Further, in this study KEGG enrichment analysis discovered that one of the multiple signaling pathways that caught our attention was the AGE-RAGE signaling pathway in diabetic complications of DOP. Advanced glycation end-products (AGE) is formed by the non-enzymatic covalent cross-linking reaction between carbohydrate and protein or fat and other biological macromolecules. The long-term existence of high blood glucose level in diabetes promotes the formation and accumulation of AGE, which can affect all tissues and organs of the body through the circulatory system (Pannucci et al. 2023). It has been shown that AGEs also play an important role in the change of bone status in high glucose environment. Studies have found that AGEs can induce apoptosis of OBs and affect bone homeostasis by mediating endoplasmic reticulum stress through proteins such as D-protein 78 and c-Jun N-terminal kinase (Suzuki et al. 2020). Okazaki K et al. found that AGEs can inhibit the differentiation of bone marrow mesenchymal stem cells (BMSC) into OBs, and bone microstructure and bone fragility are also closely related to ages (Okazaki et al. 2012). Related studies have found it play a protective role in DOP by blocking the deleterious effects of AGE-RAGE (receptor of AGE) axis (Cheng et al. 2018), which is closely related to its mediated inflammatory damage and oxidative stress damage (Wang et al. 2020; Yamagishi 2011). It has been also found that DOP patients and model animals showed significantly increased expression of AGE and inflammatory factors such as NF-κB and TNF-α in blood (Wang et al. 2020). AGE interact with RAGE and play an important role in the occurrence of OP by increasing the nuclear factor NF-κB and TNF-α of osteoclasts (OC) and OBs, leading to the disorder of bone reconstruction (Zeng et al. 2019). In this study, EPE could alleviate AGE-RAGE signaling pathway relative genes and protein expression and reduce AGEs contents, with significantly down-regulated TNF-α and NF-κB level.

Many factors lead to bone homeostasis, but the fundamental mechanism is an imbalance between osteoblasts and osteoclasts. Osteoblasts are responsible for the synthesis, secretion, and mineralization of the bone matrix, which is very important for bone formation. The previous reported that high glucose-induced could cause significantly apoptosis in OBs, with ameliorating apoptosis by regulating Bax/Bcl2 and accelerating osteogenesis in osteoblasts (Lei et al. 2023). Our results of the protein interaction network (PPI) and molecular docking showed that the core active components of quercetin, kaempferol, β-sitosterol, isorhamnetin, and stanosterol had a good binding degree with the core target proteins of BAX, BCL2, and CASP3 to improve. And EPE could significantly reduce BAX genes expression, and increased BCL2 genes expression in DOP mice.

RUNX2 and ALP are important markers of osteogenic differentiation. RUNX2 is an important transcription factor that stimulates osteogenesis and stimulating the activity of RUNX2 can regulate the osteoblast differentiation of BMMSC (Ouyang et al. 2022). ALP is a protein secreted and synthesized by osteoblast and is the main marker of osteoblast maturation and differentiation (Lei et al. 2019a, b). Study found that intervention to increase RUNX2 level and ALP activity can promote bone formation and improve osteoporosis (Lei et al. 2019a, b; Ouyang et al. 2022). Osteoclasts are the only cells in the body responsible for bone decomposition and absorption, and MMP9 is the marker gene of osteoclasts. Osteoclasts attach to old bones and secrete proteins such as MMP9, the representative marker of osteoclasts, to digest bone matrix and form absorption holes (Xiao et al. 2020). Therefore, down-regulating the expression of osteoclast-related factor MMP9 is helpful to inhibit osteoporosis (Xiao et al. 2020). Our results found that the RUNX2 and ALP expression was significantly decreased, while the MMP9 expression was significantly increased in DOP rat femur. And EPE could improve bone reconstruction by regulating ALP, RUNX2, and MMP9 expression.

## Conclusion

In conclusion, the current study utilizes a combination of bioinformatics, molecular biology, and animal experimental research to effectively demonstrate the effects and mechanisms of EPE on DOP rats. Bioinformatics analysis confirms the involvement of the AGE-RAGE in the treatment of DOP with EPE. The results of animal experiments indicate that EPE has a positive impact on improving DOP. Furthermore, EPE has been shown to this comprehensive understanding contributes valuable insights to the research on EPE treatment for DOP, offering potential for clinical applications.

Of course, there are deficiencies in this study, such as the lack of research and verification on the effective components of EPE, and the inhibition verification of AGE-RAGE pathway and other pathways. In the future, we will plan to observe the effect of EPE on DOP and AGE-RAGE pathway. By observing the effect of EPE on BMSCs osteogenic differentiation with intervene of AGEs or RAGE inhibitors to further clarify the mechanism of EPE on inhibiting AGE-RAGE signaling pathway. In addition, the major active ingredients, such as quercetin and kaempferol, will also be investigated for their effects on osteogenic differentiation, and according to the molecular docking results to verify their corresponding targets, to further explained the core role of BAX, BCL2, MMP9 and CASP3 in the treatment of DOP.

## Data Availability

No datasets were generated or analysed during the current study.

## Abbreviations

AGEs: Advanced glycation end-product
BMSC: Bone marrow mesenchymal stem cells
BS: Bone SURFace
BV: Bone volume
BV/TV: Bone volume/totalvolume
Conn-Dens: Connection density
C-T: Component-target
DOP: Diabetes osteoporosis
EPE: Epimedium brevicornum Maxim extract
MG: Model group
NG: Normal group
OC: Osteoclasts
PPI: Protein protein interaction
RAGE: Receptor of AGE
RT-PCR: Real-time polymerase chain reactions
SMI: Structure model index
Tb.N: Trabecular bone number
Tb.Sp: Trabecular spacing
Tb.Th: Trabecular thickness
TCM: Traditional Chinese medicine
TCMSP: Traditional Chinese medicine system pharmacology database and analysis platform
TSA: Tyramide signal amplification
WB: Western blotting
